# The Molecular Pathology of Odontogenic Tumors: Expanding the Spectrum of MAPK Pathway Driven Tumors

**DOI:** 10.3389/froh.2021.740788

**Published:** 2021-09-14

**Authors:** Letícia Martins Guimarães, Bruna Pizziolo Coura, Ricardo Santiago Gomez, Carolina Cavalieri Gomes

**Affiliations:** ^1^Department of Pathology, Biological Sciences Institute, Universidade Federal de Minas Gerais, Belo Horizonte, Brazil; ^2^Department of Oral Surgery and Pathology, Faculty of Dentistry, Universidade Federal de Minas Gerais, Belo Horizonte, Brazil

**Keywords:** genetic mutations, *BRAF*, *KRAS*, ameloblastoma, benign tumors, ameloblastic, odontogenic, MAPK

## Abstract

Odontogenic tumors comprise a heterogeneous group of lesions that arise from the odontogenic apparatus and their remnants. Although the etiopathogenesis of most odontogenic tumors remains unclear, there have been some advances, recently, in the understanding of the genetic basis of specific odontogenic tumors. The mitogen-activated protein kinases/extracellular signal-regulated kinases (MAPK/ERK) pathway is intimately involved in the regulation of important cellular functions, and it is commonly deregulated in several human neoplasms. Molecular analysis performed by different techniques, including direct sequencing, next-generation sequencing, and allele-specific qPCR, have uncovered mutations in genes related to the oncogenic MAPK/ERK signaling pathway in odontogenic tumors. Genetic mutations in this pathway genes have been reported in epithelial and mixed odontogenic tumors, in addition to odontogenic carcinomas and sarcomas. Notably, B-Raf proto-oncogene serine/threonine kinase *(BRAF)* and KRAS proto-oncogene GTPase *(KRAS)* pathogenic mutations have been reported in a high proportion of ameloblastomas and adenomatoid odontogenic tumors, respectively. In line with the reports about other neoplasms that harbor a malignant counterpart, the frequency of *BRAF* p.V600E mutation is higher in ameloblastoma (64% in conventional, 81% in unicystic, and 63% in peripheral) than in ameloblastic carcinoma (35%). The objective of this study was to review MAPK/ERK genetic mutations in benign and malignant odontogenic tumors. Additionally, such genetic alterations were discussed in the context of tumorigenesis, clinical behavior, classification, and future perspectives regarding therapeutic approaches.

## Introduction

Odontogenic tumors are uncommon lesions that originate from cells and tissues involved in odontogenesis and from their remnants. These tumors comprise a heterogeneous group of lesions ranging from hamartomatous ones to benign and malignant neoplasms [1]. Odontogenic tumors are classified into epithelial, mesenchymal, and mixed epithelial and mesenchymal tumors based on the odontogenic tissue they mimic [1].

Reciprocal signaling between epithelium and ectomesenchyme guides the process of tooth embryonic development, which is fully dependent on Wnt, BMP, FGF, Shh, and Eda signals [2]. The pathogenesis of odontogenic tumors is associated with alterations in components of signaling pathways. For instance, studies in the last decade have described pathogenic mutations in mitogen-activated protein kinases/extracellular signal-regulated kinases (MAPK/ERK) pathway cascade components in benign and malignant odontogenic tumors [3].

MAPK/ERK is an intracellular signaling pathway highly dependent on intracellular protein kinases. The activity of the pathway is closely related to the regulation of fundamental cellular functions such as proliferation, survival, growth, metabolism, migration, and differentiation, and alterations in this pathway could contribute to the success of neoplastic cells [4]. Although MAPK/ERK is commonly deregulated in several human cancers, the prognostic and predictive values of each mutation are context-dependent [4]. Notably, mutations in some components of this pathway, such as B-Raf proto-oncogene serine/threonine kinase *(BRAF)* and KRAS proto-oncogene GTPase *(KRAS)* genes, are oncogenic drivers but can also be identified in benign and potentially malignant conditions [3, 5, 6] and even in healthy tissues [7, 8].

Although the molecular pathogenesis of odontogenic tumors has not been completely elucidated, it is known that MAPK/ERK signaling pathway plays a role in the molecular pathogenesis of a number of them. Mutations in the MAPK/ERK pathway genes have been reported in ameloblastoma, adenomatoid odontogenic tumor, ameloblastic fibroma, ameloblastic fibrodentinoma, ameloblastic fibro-odontoma, ameloblastic carcinoma, clear cell odontogenic carcinoma, and ameloblastic fibrosarcoma [9–13].

The understanding of the core genetic changes reported in odontogenic tumors could refine their classification, aid in the diagnoses of challenging lesions, and help in the design of new targeted therapies for aggressive and/or malignant cases. Therefore, the present study has aimed to review the alterations in MAPK/ERK components reported for benign and malignant odontogenic tumors. Furthermore, we review MAPK signaling, discuss the described MAPK/ERK genetic alterations in the context of tumorigenesis, biological behavior, entity classification, and future perspectives regarding targeted therapy.

The search strategy included the terms “odontogenic tumor,” “odontogenic tumour,” “MAPK,” “MAPK/ERK,” “mutation,” “FGFR,” “BRAF,” “KRAS,” “NRAS,” “HRAS,” “ameloblastoma,” “adenomatoid odontogenic tumor,” “ameloblastic fibroma,” “ameloblastic fibrodentinoma,” “ameloblastic fibro-odontoma,” “ameloblastic carcinoma,” “clear cell odontogenic carcinoma,” and “ameloblastic fibrosarcoma” which were connected with appropriate Boolean operators “AND” and “OR”. Important papers from the background knowledge and the reference list of the studies were also added. All studies that used molecular techniques and/or immunohistochemistry to assess MAPK/ERK mutations in odontogenic tumors were included.

## MAPK Signaling Pathways

The MAPK signaling pathways are master regulators of multiple cellular responses whose activation is triggered by a wide variety of extracellular signals [4]. Transmembrane receptors can recognize stimuli, including hormones, growth factors, mitogens, and inflammatory cytokines, and transmit signals from the extracellular membrane to the nucleus through phosphorylation cascades which then regulate fundamental cellular functions [4].

Eukaryotic cells have at least seven MAPK pathways named according to their final regulatory proteins [14]. They are classified as conventional or atypical mainly based on the activation mechanisms [14]. Each MAPK pathway has a specific mode of activation and downstream functions due to variations in protein expression, subcellular compartmentalization, interaction in protein complexes (*scaffolds proteins*) partners, and substrate-targeting mechanisms leading to distinct cellular responses [4]. Among the conventional MAPK pathways, extracellular regulated kinases (ERK1/2), Jun NH2 terminal kinases (JNK1/2/3), and p38 (p38 α/β/γ/δ) are the most well-characterized groups [4, 15].

**Figure 1:**
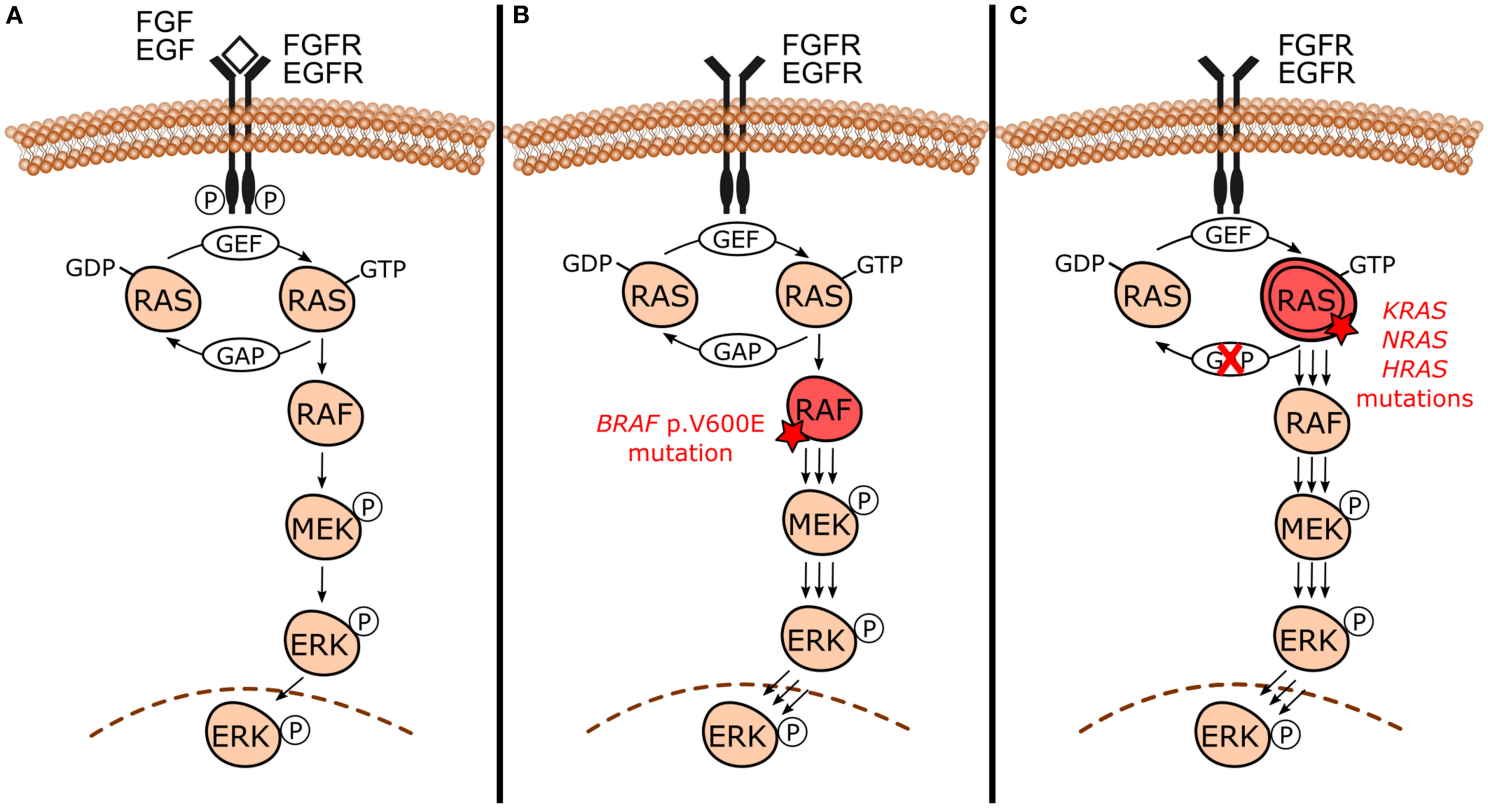
Canonical mitogen-activated protein kinases/extracellular signal-regulated kinases (MAPK/ERK) signaling pathway under normal circumstances and in the presence of activating mutations. **(A)** Canonical RAS-RAF-MEK-ERK cascade and its normal activation by external signals (e.g., FGF and EGF growth factors) binding to receptor tyrosine kinases [e.g., fibroblast growth factor receptor (FGFR) and epidermal growth factor receptor (EGFR)]. This interaction triggers signaling through RAS-GTP, RAF, MEK, and ERK culminating with the action of phosphorylated ERK in its substrates and the regulation of cellular biological functions. In the absence of external stimulus, active RAS-GTP switches to its inactive form RAS-GDP by hydrolysis due to GAPs action. **(B)** In the presence of *BRAF* p.V600E mutation, BRAF constitutively activates MAPK/ERK signaling even in the absence of growth factors and dimerization with RAS, sustaining MAPK/ERK signaling. **(C)** Activating mutations in *RAS* genes (*KRAS, NRAS*, and *HRAS)* lead to unbalance between inactive and active RAS forms toward the active state (RAS-GTP) either by reducing GTP hydrolysis or by increasing the rate of GTP loading. This mechanism constitutively activates MAPK/ERK signaling even without external stimulus, sustaining its signals.

Even though each MAPK pathway is unique, they share the presence of three evolutionarily conserved effectors, which are sequentially acting protein kinases generically known as MAPKKK, MAPKK, and MAPK [15–17]. In the canonical MAPK/ERK signaling pathway (Figure 1A), specifically in the RAS-RAF-MEK-ERK cascade, it is notable that these intracellular effectors correspond to RAF (MAPKKK), MEK1/2 (MAPKK), and ERK1/2 (MAPK) proteins. They are further upregulated by the RAS GTPases family (RAS). RAS proteins are located at the inner surface of the cell membrane and maintain balance through cycling between their inactive and active states through binding to guanosine diphosphate (GDP) and guanosine triphosphate (GTP), respectively. The conversion from its stable and quiescent cytoplasmic form to the active form is catalyzed by guanine nucleotide exchange factors (GEFs) through reversible phosphorylation reaction, therefore leading to downstream pathway activity. GTP-loaded RAS recruits and interacts directly with RAF protein, promoting its activation through complex processes that culminate in dimerization of the RAS-RAF binding domain. Activated RAF phosphorylates MEK within the activation segment and promotes the recruitment and activation of ERK through phosphorylation on both threonine and tyrosine sites. Once activated, ERK phosphorylates RSK, MSK, and MNK cytoplasmic targets, and translocates to the nucleus where it finally has access to nuclear substrates. Transcription factors, other proteins which include kinases and phosphatases, and cytoskeletal elements are some nuclear targets involved in MAPK/ERK cascade. The ERK phosphorylation-induced activity usually results in the activation of these substrates. However, it can degrade and inhibit them in some cases. Usually, after suspension of the external stimulus, the active form of RAS switches to the inactive state of RAS by hydrolysis of GTP due to the direct interaction of GTPase-activating proteins (GAPs) with RAS-GAP complex formation. This complex formation, followed by dephosphorylation reactions mediated by MAPK phosphatases and negative feedback, are crucial components involved in the inactivation of the pathway [4, 15, 18, 19].

MAPK/ERK pathway coordinates six essential physiological processes in response to extracellular signals, including cell proliferation, survival, growth, metabolism, migration, and differentiation [4, 15, 18, 19].

Generally speaking, MAPK signaling cascades appear to have simple and linear biochemical actions, with the unidirectional activity of protein kinases. Nevertheless, the pathway is highly intricate and the complexity and pleiotropism of the MAPK/ERK pathway are genuinely linked to the multiple subsets of ERK substrates. Diversity of MAPK components isoforms, duration, and intensity of signals, protein localization in different cell compartments, scaffold proteins, and cross-talk are the mechanisms that enhance the range of the regulatory activity of the MAPK/ERK pathway [4, 15, 18, 19].

Alterations in MAPK/ERK components in the different tiers of the cascade culminate in the constitutive activation of the pathway. This leads to abnormal crucial cellular functions that can significantly contribute to the evolutionary advantages of neoplastic cells when compared to surrounding cells [20, 21]. Indeed, genetic studies have shown that MAPK/ERK is commonly deregulated in many human diseases, including human cancers and RASophaties [4, 15].

Although extensive molecular analyses have focused on cancer molecular investigations, recent advances in genome screening and other gold-standard molecular techniques have aided to elucidate the molecular basis in a non-malignant context. Benign and potentially malignant lesions can harbor oncogenic mutations once considered exclusive of cancer. Furthermore, these mutations sometimes occur at a higher frequency in the benign lesions than in their corresponding malignant counterparts [3, 5, 6].

Odontogenic tumors have been shown to harbor high frequencies of both *BRAF* and *RAS* mutations [9–13]. The *BRAF* gene encodes the BRAF protein, which is a RAS-regulated cytoplasmic serine-threonine kinase that activates the MAPK/ERK signaling pathway by phosphorylating MEK [4, 22]. The gene *BRAF* is the only member of the *RAF* gene family to be frequently activated by mutations in human neoplasms [23]. The missense *BRAF* mutation that results in a valine (V) to glutamic acid (E) substitution at codon 600 (*BRAF* p.V600E) constitutively activates MAPK/ERK pathway [21] (Figure 1B). Aside from stimulating MAPK/ERK signaling, *BRAF* p.V600E induces cell proliferation and is capable of promoting transformation, which collectively supports its classification as an oncogene [24]. In an experimental model using Braf p.V600E knock-in mouse, expression of the mutation in all tissues lead to embryonic lethality [25]. Therefore, it is unlikely that *BRAF* p.V600E mutation is compatible with life in humans. Since 2002, *BRAF* pathogenic mutations frequencies and their implications have been studied in a wide variety of tumor types wherein the majority specifically involve the *BRAF* p.V600E mutation [26, 27]. *BRAF* gene mutation has been identified in about 6% of human cancers [27], including melanomas (40–50%) [27–29], thyroid cancers (10–70%) [27, 30], colorectal cancers (~10%) [27, 31, 32], and non-small cell lung cancer (3–5%) [27, 33].

*KRAS*, NRAS proto-oncogene GTPase *(NRAS)*, and HRas proto-oncogene GTPase *(HRAS)* are members of RAS proto-oncogenes family *(RAS)* and they play an important role in human cancers [34]. These genes encode RAS GTPases, which are upstream activators of MAPK/ERK and act by interacting and activating RAF proteins. *RAS* mutations occur in 30% of human malignant neoplasms and *KRAS* is the most frequent mutated oncogene. *KRAS* codon 12 mutations correspond to nearly 90% of all *KRAS* mutations in pancreatic, colorectal, and lung human cancers [34]. *RAS* mutations constitutively activate MAPK/ERK pathway, as shown in Figure 1C.

Interestingly, some studies have also reported somatic oncogenic mutations in normal endometrium and breast cells of healthy women [7, 8]. The study of Coura and colleagues (2020) investigated the presence of *KRAS* p.G12V and p.G12R and *BRAF* p.V600E mutations in normal odontogenic tissue remnants, specifically in dental follicles associated with unerupted teeth, as an attempt to uncover early oncogenic mutations that could lead to odontogenic tumors tumorigenesis. However, these specific *KRAS* and *BRAF* mutations have not been detected in any of the investigated samples [35]. Therefore, despite the presence of MAPK/ERK oncogenic mutations in odontogenic tumors, the role of such mutations play in the pathogenesis of these lesions remains to be clarified.

### MAPK/ERK Signaling Pathway Cross-Talk

Cross-talk between MAPK cascades and other cellular regulatory pathways can extensively influence MAPK/ERK signaling by dynamic and complex interactions [4, 36].

Illustrating such phenomena, cross-talk between MAPK/ERK and PI3K/AKT/mTOR pathways at different stages of signal propagation can amplify key target regulatory protein activities through cross-activation and/or substrate convergence [4, 36]. MAPK/ERK regulates PI3K, TSC, and mTOR activity by activating PI3K and mTORC1 and inhibiting TSC2 [4, 36]. In this case, an important positive loop involves PI3K activation and GAB docking proteins phosphorylation. Once phosphorylated, GAB interferes in RAS-GAP complex binding, decreasing RAS inactivation and leading to positive ERK upstream regulation [36]. Moreover, ERK, RSK, AKT, and S6K protein kinases, and the components of MAPK/ERK and PI3K/AKT/mTOR pathways, often phosphorylate the same substrate including FOXO and c-Myc transcription factors [4, 36].

It is also known that the WNT/β-catenin signaling pathway interferes in MAPK/ERK regulation. The active GSK3β in the destruction complex (binding to β-catenin, APC, and AXIN) phosphorylates RAS, which is consequently degraded. Aberrant activation of the WNT/β-catenin pathway leads to excessive dissociation of destruction complex with consequent accumulation of β-catenin and RAS proteins in the cytoplasm. This promotes the β-catenin translocation to the nucleus and facilitates MAPK/ERK downstream activity by increasing RAS availability [37, 38]. In that case, mutual deregulation in MAPK/ERK and hyperactivation of WNT/B-catenin seems to cooperatively act triggering tumorigenesis [37, 38].

### MAPK/ERK Pathway in Odontogenesis

Interactions between epithelial and ectomesenchymal cells govern tooth development during odontogenesis. The molecular mechanisms associated with odontogenesis involve intracellular signaling cascades, including MAPK, Hedgehog, and Wnt pathways, and alterations in these pathways have been associated with the pathogenesis of odontogenic lesions [2, 3]. It is beyond the scope of this review to discuss the Hedgehog and Wnt pathways and the molecular pathogenesis of odontogenic lesions related to them. Thereby, we focused on the prototypical MAPK/ERK signaling pathway and the odontogenic tumors associated with its disturbance.

MAPK/ERK signaling plays a role in odontogenesis [39]. Unlike humans, mice are monophyodonts and have intrinsic differences from humans. However, mice dentition is a useful model to study the mechanisms related to human dental development [39, 40]. Supporting a role for MAPK/ERK signaling in odontogenesis, mice carrying deletions in Sprouty genes, which encode negative regulators of MAPK/ERK pathway, have hyperactive MAPK/ERK pathway signaling and supernumerary teeth [39, 41]. Similarly, ribosomal protein S6 kinase *(RSK)* mutant mice develop supernumerary teeth and alteration in dental shape patterns [39, 40]. RSKs are protein kinases that act downstream of the MAPK/ERK cascade and seem to have a feedback inhibitory effect on MAPK/ERK signaling [39, 40]. It is notable that the mutations in *RSK2* cause Coffin-Lowry syndrome (OMIM #303600), in which dental anomalies can be present [40].

In addition, experimental data support the association of MAPK/ERK with odontogenic tumors tumorigenesis through *RAS* genes. Notably, Hras-G12V mutant mice show defects in the differentiation and proliferation of ameloblasts and their precursors [42, 43], while Hras transgenic mice develop jaw tumors consistent with odontogenic tumors [44–46]. Further supporting a role for MAPK/ERK signaling in odontogenesis, KRAS, RAF1, MEK1, and ERK1/2 protein expressions have been detected in human tooth germs by immunohistochemistry [47].

The fibroblast growth factor receptor 1 (FGFR1), which is a receptor whose stimulation activates MAPK/ERK pathway, has a strong expression in mouse odontoblasts, in addition to FGFR2 (isoform IIIb) in ameloblasts. This suggests that the fibroblast growth factors (FGFs) have a role in the regulation of their differentiation and secretory functions [48]. Moreover, the expression patterns of different FGFs in dental epithelium and mesenchyme are dynamic, supporting the existence of regulatory signaling cascades between FGFs in both these tissues during odontogenesis [49]. Another receptor whose stimulation activates MAPK/ERK pathway is the epidermal growth factor receptor (EGFR). There is consistent evidence that EGFR expression during odontogenesis has an important role during this developmental process [50].

Collectively, the above-mentioned studies support a role for MAPK/ERK signaling pathway in odontogenesis regulation, and disturbance in this pathway may lead to either odontogenesis impairment or can give rise to odontogenic tumors in animal models.

## MAPK/ERK Mutations in Odontogenic Tumors

**Figure 2:**
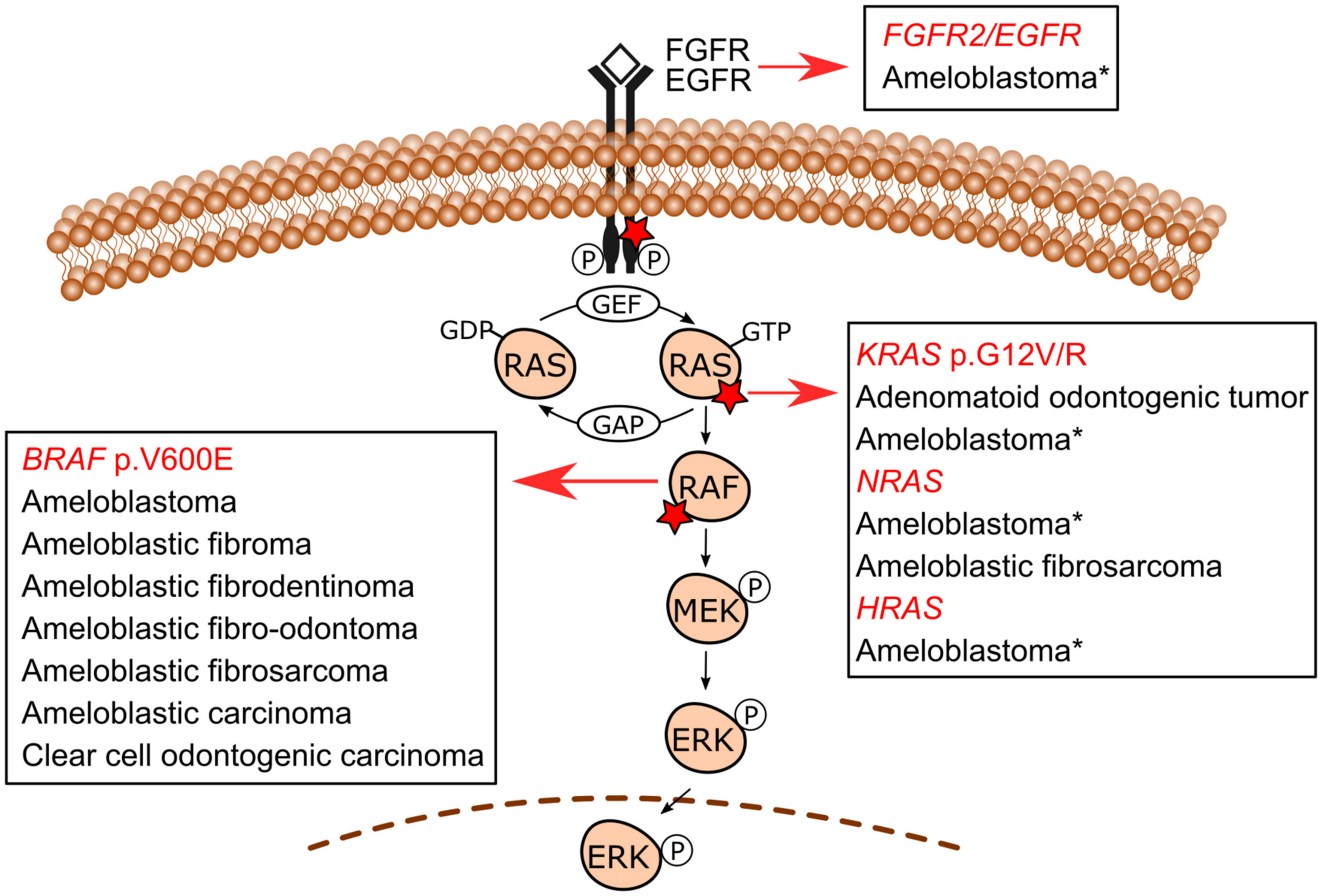
MAPK/ERK signaling pathway mutations in odontogenic tumors. *BRAF* p.V600E is the most common mutation in ameloblastomas, followed by *KRAS* (mostly p.G12R), *NRAS, HRAS*, and *FGFR2* mutations reported in a few *BRAF* wild-type cases. Additionally, an *EGFR* mutation has also been reported in one ameloblastoma case. These less commonly reported mutations in ameloblastomas are indicated with an asterisk (*). Ameloblastic fibromas, ameloblastic fibrodentinomas, ameloblastic fibro-odontomas, ameloblastic fibrosarcoma, ameloblastic carcinomas, and clear cell odontogenic carcinoma (a single case) also carry *BRAF* p.V600E mutation. An *NRAS* mutation has also been reported in one case of ameloblastic fibrosarcoma in a mutually exclusive manner with *BRAF* p.V600E. Adenomatoid odontogenic tumors are characterized by frequent *KRAS* codon 12 (either p.G12V or p.G12R, and in a single case p.G12D) driver mutations, which occur in approximately 70% of cases. Detailed information on the studies reporting these mutations is shown in Table 1.

The use of molecular pathology techniques has led to the identification of MAPK/ERK-related gene mutations in specific odontogenic tumors. Initially, such mutations have been uncovered in benign epithelial odontogenic tumors wherein *BRAF* and *KRAS* mutations were revealed in ameloblastomas and adenomatoid odontogenic tumors, respectively [9, 11]. In recent studies, mixed odontogenic tumors and malignant odontogenic tumors have also been included in the spectrum of MAPK pathway-driven tumors. Figure 2 shows the MAPK/ERK genes for which mutations have been described in odontogenic tumors. Table 1 summarizes the frequency of mutations in each tumor.

**Table 1:** Summary of MAPK/ERK mutations and their frequencies in odontogenic tumors.

**Odontogenic tumors**	**Mutations in MAPK/ERK pathway genes**	**Frequency of each mutation^#^**	**References**
**Benign epithelial odontogenic tumors**			
Conventional Ameloblastoma	*BRAF* p.V600E	64% (478/746)^a^	[9], [10], [51], [52], [53]^e^, [54], [55], [56], [57], [58], [59], [60], [61], [62], [63], [64], [65], [66]^f^, [67]^f^, [68]^f^, [69]^f^, [70]^f^, [71]^f^
	*KRAS* ^*^ ^b^	7.2% (13/180)	[51], [52], [55]^g^, [62], [72]
	*NRAS* ^*^ ^c^	5.2% (8/155)	[52], [54], [55]^g^, [58]
	*HRAS* ^*^ ^c^	4% (6/151)	[52], [55]^g^, [58]
	*FGFR2* ^*^ ^d^	11.3% (14/124)	[51], [52], [58], [72]
	*EGFR* ^*^	1.6% (1/62)	[55]^g^
Unicystic ameloblastoma	*BRAF* p.V600E	80.7% (109/135)	[10], [55], [58]^h^, [60], [62], [63], [64], [68]^f^, [69]^f^, [70]^f^, [71]^f^ [73]
Peripheral ameloblastoma	*BRAF* p.V600E	62.5% (10/16)	[54], [55], [62], [69]^f^, [71]^f^
	*NRAS* p.Q61R^*^	33.3% (1/3)	[54]
Adenomatoid odontogenic tumor	*KRAS* p.G12V	42.9% (24/56)	[11], [12], [74]
	*KRAS* p.G12R	34% (16/47)	[12], [74]
	*KRAS* p.G12D	11.1% (1/9)	[74]
**Benign mixed epithelial & mesenchymal odontogenic tumors**			
Ameloblastic fibroma	*BRAF* p.V600E	45.8% (11/24)	[13], [52], [53], [63]
Ameloblastic fibrodentinoma	*BRAF* p.V600E	60% (3/5)	[13], [52]
Ameloblastic fibro-odontoma	*BRAF* p.V600E	34.6% (9/26)	[13], [53], [63]
**Odontogenic sarcomas**			
Ameloblastic fibrosarcoma	*BRAF* p.V600E	70% (7/10)	[13], [75]
	*NRAS* p.Q61K^*^	14.3% (1/7)	[75]
**Odontogenic carcinomas**			
Ameloblastic carcinoma	*BRAF* p.V600E	35.3% (6/17)	[10], [53], [63]
		100% (5/5)^**^	[76]^**^
Clear cell odontogenic carcinoma	*BRAF* p.V600E	100% (1/1)	[10]

a *Individual frequency per study ranged from 30 to 90%, except for two studies that have reported mutations in all samples probably due to limited sample size [2/2 [64] and 4/4 [65]]*.

b *In eight out of 13 (61.5%) cases the mutation was KRAS p.G12R and in one sample KRAS p.L56_G60dup. For the other cases, the specific mutation was not reported*.

c *With regard to NRAS, p.Q61R was the most frequent mutation, occurring in approximately 50% of the eight NRAS mutation-positive samples, and NRAS p.Q61K was also reported. With regard to HRAS mutations, HRAS p.Q61R was the most frequent one, occurring in 3/6 (50%) of HRAS mutation-positive samples, HRAS p.G12S and p.Q61K were reported in one case each, and the other one was not specified*.

d *FGFR2 p.C382R accounted for 9/14 (64%) of cases (FGFR2 Uniprot P21802-1; isoform 1, FGFR2IIIc is the canonical sequence). FGFR2 p.N549K, p.Y376C, and p.V396D were detected in one case each. FGFR2 p.V395D was also reported. Based on the published information we could not ascertain which specific mutation corresponds to the 14th case. Gültekin et al. [55] reported one FGFR mutation in 1/62 cases, however, it is not clear if it occurred in FGFR2. Therefore, we opted to not include this data for FGFR2*.

e *The authors reported BRAF p.V600E mutation in five unicystic cases, but they did not specify the total number of unicystic cases assessed. Therefore, we opted to include all the mutation-positive samples (14/19) in the conventional ameloblastoma count*.

f *These studies based their results on BRAF p.V600E immunohistochemistry only. For studies that performed molecular screening in addition to immunohistochemistry and had discordant results, we considered the molecular results*.

g *In the paper by Gültekin et al. [55], it is not clear if any KRAS, NRAS, HRAS, and EGFR mutations were detected in variants other than conventional ameloblastoma. Therefore, we considered the total number of assessed samples (n = 62), to calculate the frequency of KRAS, NRAS, HRAS, and EGFR mutations*.

h *Considering only the mandibular location, The study of Heikinheimo et al. [58] described BRAF p.V600E in 29/31 (93.5%) of unicystic ameloblastomas*.

* *Mutually exclusive with BRAF p.V600E, except for one case harboring FGFR2 p.C382R and BRAF p.V600E*.

** *This study reported BRAF p.V600E mutation in all five ameloblastic carcinoma samples evaluated and this frequency was much higher than reported by previous studies, and therefore we did not add the results to the other ones when calculating mutation frequency*.

# *Different detection methods were used in the studies to assess mutations, including allele-specific qPCR, next-generation sequencing, direct sequencing as well as immunohistochemistry*.

The following sections addressed the main genetic alterations reported for ameloblastomas, mixed odontogenic tumors, odontogenic carcinomas, where *BRAF* p.V600E mutation is the most important one, and adenomatoid odontogenic tumors which are characterized by *KRAS* mutations.

### Ameloblastoma

Ameloblastoma is a locally aggressive benign epithelial odontogenic tumor occurring mainly in the posterior mandible. Although benign, this tumor can behave aggressively and recur. Surgical approaches to remove this tumor often lead to facial deformity and morbidity [1]. Ameloblastomas are differently categorized into ameloblastoma (conventional), unicystic type, extraosseous/peripheral type, and metastasizing ameloblastoma, with distinct biological behaviors [1].

In 2014, the pioneer study by the group of Heikinheimo reported for the first time recurrent activating *BRAF* p.V600E mutations in ameloblastomas [9]. Considering that MAPK/ERK signaling can be activated by stimulation of transmembrane receptors, including EGFR, and that overexpression of EGFR had previously been reported in ameloblastomas, when the investigators detected ameloblastoma cultured cells resistant to EGFR-targeted inhibition they screened these cells for BRAF p.V600E mutation [9]. After detecting the mutation in this index case, the study of Kurppa et al. (2014) detected *BRAF* p.V600E mutation in 62.5% (15/24) of ameloblastoma samples through Sanger sequencing [9]. In addition, the study of these authors demonstrated the utility of the *BRAF* p.V600E-specific monoclonal antibody VE1 to detect the *BRAF* p.V600E mutation in ameloblastomas by immunohistochemistry [9].

The study of Sweeney et al. carried out targeted next-generation sequencing (NGS) and/or Sanger sequencing and VE1 immunohistochemistry where they detected *BRAF* p.V600E mutation in 43% (12/28) of ameloblastoma samples [51]. Additionally, they detected another *BRAF* variant (p.L597R) in one sample [51]. The study of Brown et al. performed a combination of allele-specific PCR, NGS gene panel, and Sanger sequencing and showed *BRAF* p.V600E in 62% (31/50) of ameloblastoma cases [52]. They further reported the presence of *BRAF* p.V600E mutation in 67% (23/34) of ameloblastomas by using VE1 immunohistochemistry in cases not suitable for molecular evaluation [52]. Following these pieces of research, several studies have also assessed the presence of *BRAF* p.V600E in conventional ameloblastoma, either by molecular techniques or a combination of mutation screening and immunohistochemistry, with mutation frequencies varying from 55 to ~90% [10, 53–63]. In two studies with limited sample numbers, the mutation was detected in all samples [2/2 [64] and 4/4 [65]]. The frequency of *BRAF* p.V600E mutation in studies that have assessed the mutation only using immunohistochemistry varied from 33 to 79% [66–71]. Notably, although immunohistochemical expression of BRAF p.V600E has been used as a surrogate of *BRAF* p.V600E mutation, results must be interpreted with caution since there are false-positive cases detected by immunohistochemistry and not confirmed by molecular technique [73] as well as false-negative cases [63].

There is no consensus on the association of *BRAF* p.V600E mutation with ameloblastoma clinicopathological features, including location, age, histology, as well as clinical behavior. In the studies conducted by Sweeney et al. and Gültekin et al., they suggested the predominance of *BRAF* p.V600E ameloblastomas in mandible when compared to *BRAF* wild-type tumors [51, 55]. The studies of Brown et al., Bonacina et al., and Kelppe et al. have also suggested *BRAF* p.V600E positive cases mostly locate in the mandible and occur at earlier ages [52, 62, 69]. In agreement with that, the study of Gültekin et al. has also reported that *BRAF* p.V600E ameloblastoma cases occur at a much younger age than *BRAF* p.V600E wild-type ones [55]. Additionally, *BRAF* mutations have been reported by some authors to predominantly occur in non-plexiform histologic type ameloblastoma [51, 55, 71], while conversely others reported *BRAF* mutations predominance in plexiform type [67]. It is worth mentioning that in this last study [67], the authors have assessed the mutation using immunohistochemistry as a surrogate marker for the mutation. However, they used a different antibody clone other than VE1. Nonetheless, most studies reported recurrent *BRAF* p.V600E mutation regardless of histological type [9, 10, 56, 60, 66], mandibular/maxillary location [10, 56, 66], and the age of patients [9].

The association between the presence of *BRAF* p.V600E and ameloblastoma aggressiveness is unclear. While some studies had reported earlier recurrence and increased recurrence rates in *BRAF* wild-type tumors [52, 55, 58], others had observed a more aggressive behavior with poor disease-free survival and higher recurrence rate in ameloblastomas harboring *BRAF* p.V600E mutation; in these cases, assessed by immunohistochemistry only [66, 67]. In recent times, no difference has been found in relapse-free intervals between *BRAF* p.V600E positive and wild-type ameloblastoma cases [62].

Mutations in 28 genes have been assessed by an NGS targeted panel, in addition to smoothened frizzled class receptor *(SMO)* mutations, assessed by Sanger sequencing [55]. Mutations have been detected in approximately 90% of the ameloblastomas, comprised of conventional, unicystic, and peripheral cases, with *BRAF* p. V600E corresponding to 60% of mutation-positive cases [55]. *SMO* mutations have been detected in 14% of cases, in a mutually exclusive pattern with *BRAF* mutations. Additionally, *NRAS, HRAS*, and *EGFR* somatic mutations have been detected in one case each, mutually exclusive with *BRAF* and *SMO* mutations. In a few cases, *BRAF* or *SMO* mutations co-occurred with mutations in other genes. Importantly, the authors reported high recurrence rate in ameloblastomas harboring multiple gene mutations (higher mutational burden) detected either by targeted NGS or Sanger sequencing. At the same time, a low risk for relapse was observed in tumors with a single *BRAF* mutation [55]. It is notable that the presence of two or three gene mutations, including somatic mutations in *KRAS, PIK3CA, PTEN, FGFR, CDKN2A*, and *CTNNB1* in the background of either *BRAF* or *SMO* mutation-positive ameloblastomas, exclusively occurred in solid/conventional tumors [55]. Further studies may help to clarify if these associations between tumor mutation burden and aggressiveness hold.

The study of Diniz and co-workers in 2015 tested whether the *BRAF* p.V600E mutation was a signature of conventional ameloblastoma or if unicystic lesions also harbored this mutation. *BRAF* p.V600E was detected in 83% (5/6) of unicystic ameloblastomas [10]. In the later study of Heikinheimo et al., they reported *BRAF* p.V600E mutation in 94% (29/31) of mandibular unicystic ameloblastomas and higher homogeneity of mutations than that of conventional ameloblastomas, since rare samples carried a mutation other than *BRAF* [58]. The mutational screening of *BRAF* by molecular assays has been shown to be useful for the differential diagnosis between unicystic ameloblastomas and odontogenic cysts [64, 73]. Furthermore, a consistent homogeneous profile for *BRAF* mutational status has been shown in unicystic ameloblastomas, with *BRAF* p.V600E detection by allele-specific qPCR in all areas with different histological appearances from the same neoplasm, including those areas resembling other odontogenic lesions [64]. Other studies that reported *BRAF* p.V600E mutation in the unicystic variant by molecular screening and/or immunohistochemistry are listed in Table 1.

A small number of studies have assessed genetic mutations in peripheral ameloblastomas [54, 55, 62, 69, 71]. The *BRAF* p.V600E was also the most common mutation among them, and it was detected in 62.5% (10/16) of cases [54, 55, 62, 69, 71]. Additionally, single somatic mutations in *NRAS* [54] and *SMO* [55] have also been detected in one peripheral ameloblastoma sample each in a mutually exclusive manner with the *BRAF* mutation.

Mutually exclusive and less common mutations affecting other MAPK-related genes, such as *KRAS, NRAS, HRAS*, and *FGFR2* (a receptor whose stimulation activates MAPK/ERK pathway) have been reported in *BRAF* wild-type ameloblastomas [51, 52, 54, 55, 58, 62, 72]. It is notable that the *KRAS* p.G12R mutation, which occurs in adenomatoid odontogenic tumors [12], has been reported in 4/50 (8%) [52] and 4/28 (14%) [51] of *BRAF* wild-type ameloblastoma cases. Furthermore, in addition to the above-mentioned mutations in MAPK pathway genes, somatic *EGFR* mutation has been detected in one single case of maxillary ameloblastoma [55]. Mutations in *FGFR2* have been described in 4/28 (14%) [51], 3/50 (6%) [53], and 2/39 (5%) [58] of *BRAF* wild-type conventional ameloblastomas. The study of Bartels et al. assessed *BRAF* wild-type ameloblastomas by targeted NGS and identified *FGFR2* mutations in 4/7 (57%) of them, including one *FGFR2/TP53/PTEN*-triple-mutant case [72]. Additionally, increased proliferation through MAPK/ERK activation of ameloblastomas cultured cells has been shown after treatment with FGF ligands [77], reinforcing the role of FGF signaling in the tumorigenesis of ameloblastomas.

Other cancer-associated mutations not directly involved in the MAPK pathway have also been detected in ameloblastomas [51, 52, 54, 55, 57, 58, 72, 78]. Among them, *SMO* mutations have been detected in between 13 and 39% of ameloblastomas, occurring in a mutually exclusive pattern with *BRAF* p.V600E [51, 52, 55]. *SMO* mutations often occur along with an additional *RAS* family or *FGFR2* mutation [51, 52, 55]. Interestingly, the study of Sweeney et al. proposed site-specific *BRAF* and *SMO* mutations in mandible and maxilla, respectively [51]. This finding was later supported by the study of Gültekin et al. in a larger cohort [55]. However, the site-specificity of these mutations has not been confirmed by other groups [10, 58]. Aside from the *SMO* mutations, *APC* mutations have been detected in 3/6 ameloblastomas [78], and *CTNNB1, PIK3CA, SMARCB1, PTEN, CDKN2A* mutations have been reported in a few cases and tend to occur in a non-mutually exclusive manner with *BRAF* p.V600E mutation and with each other [52, 54, 55]. Protein products of some of these genes, e.g., APC, β -catenin encoded by *CTNNB1*, and PI3K encoded by *PI3KCA*, are components of pathways that cross-talk with MAPK/ERK, as discussed in the “MAPK/ERK signaling pathway cross-talk” topic.

Recently, two studies performed whole-exome sequencing to assess coding mutations in ameloblastomas and reported uncommon mutations in several other genes occurring in the background of *BRAF* p.V600E mutation [57, 65]. The study of Guan et al. reported *BRAF* p.V600E mutation in 82% (9/11) of samples and proposed intratumor heterogeneity in ameloblastomas based on different variant allele frequencies (VAF) of *BRAF* p.V600E and other detected mutations [57]. Mutations in the *CDC73* gene, which encodes parafibromin, have been detected in 2/11 samples [57]. It is worth mentioning that *CDC73* germline mutation causes hyperparathyroidism-jaw tumor syndrome (OMIM #145001), in which ossifying fibromas may occur. The study of Shi et al. suggested a “two-hits” mechanism contributing to ameloblastoma tumorigenesis, with *BRAF* p.V600E (detected in all four assessed samples) corresponding to the first hit and mutations in genes belonging to the gene network of cell proliferation, such as *HSAP4*, corresponding to the second hit [65]. However, further investigations in larger cohorts and using other methodologies for evaluating tumor clonality and the role of the detected mutations in ameloblastoma development, if any, are needed to confirm these findings. Currently, the evidence is still insufficient to draw any conclusions about further mutations other than *BRAF* p.V600E participating as drivers of ameloblastoma pathogenesis.

### Benign Mixed Epithelial & Mesenchymal Odontogenic Tumors

The *BRAF* p.V600E mutations have been reported in benign mixed odontogenic tumors [13, 52, 53, 63], namely ameloblastic fibroma [13, 52, 53, 63], ameloblastic fibrodentinoma [13, 52], and ameloblastic fibro-odontoma [13, 53, 63].

Collectively, these studies reported *BRAF* p.V600E mutations in 45.8% (11/24) of ameloblastic fibromas [13, 52, 53, 63], 60% (3/5) of ameloblastic fibrodentinomas [13, 52], and 34.6% (9/26) of ameloblastic fibro-odontomas [13, 53, 63], whereas all nine odontoma samples investigated were wild-type for *BRAF* p.V600E [13, 63]. These results suggested that at least a subset of ameloblastic fibromas, ameloblastic fibrodentinomas, and ameloblastic fibro-odontomas are pathologic entities distinct from odontomas. However, this subject is still debatable.

It was suggested that the *BRAF* p.V600E mutation was limited to the epithelial component of the four mutant cases of mixed tumors which had the two components tested separately, i.e., 1/1 mutant ameloblastic fibroma and 3/3 mutant ameloblastic fibro-odontomas [53]. However, our group has recently dealt with this issue using a straightforward methodology. We have subjected mixed odontogenic tumor samples to laser capture microdissection before molecular testing in an attempt to avoid epithelial-mesenchymal cross-contamination and using allele-specific quantitative PCR, a high sensitivity mutation detection assay, we detected the *BRAF* p.V600E mutation in the mesenchymal component of all mutation-positive mixed odontogenic tumors (9/9) [13]. Notwithstanding, *BRAF* p.V600E positive status was additionally detected in the epithelial components in two cases, which are one ameloblastic fibroma and one ameloblastic fibro-odontoma [13]. While this positivity in the epithelial and mesenchymal components of these two samples may have occurred due to cross-contamination and detected using a high-sensitivity molecular technique, it may also represent the true mixed nature of such lesions.

### Odontogenic Sarcomas

The molecular pathogenesis of odontogenic sarcomas, namely ameloblastic fibrosarcoma, has been explored recently [13, 75]. The *BRAF* p.V600E mutation has been detected in 71% (5/7) [75] and 67% (2/3) [13] of cases. Consistent with the histopathologic features of the ameloblastic fibrosarcoma, in which malignancy is restricted to the mesenchymal component, the *BRAF* p.V600E mutation was restricted to the sarcomatous areas of the two mutant ameloblastic fibrosarcoma cases tested in our cohort [13]. The mutation was further detected in the benign mesenchymal component of the ameloblastic fibroma-like area in one of the ameloblastic fibrossarcoma positive cases [13]. This finding reinforces the malignant transformation from a previous ameloblastic fibroma in some cases [13]. In addition to *BRAF* mutations, *NRAS* p.Q61K has been detected in a *BRAF* wild-type ameloblastic fibrosarcoma sample [75].

Although the rarity of this tumor precludes extensive knowledge about its molecular pathology, the current results could support the role of the MAPK/ERK pathway in its pathogenesis and pave the way for further investigation on targeted therapy.

### Odontogenic Carcinomas

Ameloblastic carcinomas, the malignant counterpart of ameloblastomas, also harbor *BRAF* p.V600E mutations, but with reported frequencies varying from 25 to 40% [10, 53, 63]. Such frequencies are lower than those reported for the same mutations for ameloblastomas (64% in conventional, 81% in unicystic, and 63% in peripheral), as shown in Table 1. It is worth noting that the lower frequency of *BRAF* p.V600E mutation in ameloblastic carcinomas compared with ameloblastomas is similar to what is observed in other benign tumors and their malignant counterparts [6]. For instance, *BRAF* p.V600E mutation is detected in approximately 80% of benign melanocytic nevi, 60% of dysplastic nevi, and only in 40–45% of melanomas, suggesting that the functional effects of the mutation are context-dependent [6]. Additionally, oncogene-induced senescence may limit the proliferation status of nevi in the tumorigenic process [79]. However, *BRAF* p.V600E mutation has been recently reported in 5/5 ameloblastic carcinoma cases submitted to molecular screening [76]. These results could encourage targeted therapy as a new direction in the future.

Mutations in other genes which are not related to MAPK/ERK, such as *TP53, CTNNB1*, and *APC*, have also been reported in ameloblastic carcinomas [72, 78, 80]. The detection of *TP53* mutation in the malignant area of a tumor arising from preexisting ameloblastoma [80] and observation of *TP53* and *CTNNB1* in a *BRAF* p.V600E wild-type ameloblastic carcinoma [72] suggest that mutations in these genes might play a role in the malignant transformation process of ameloblastomas.

Odontogenic carcinoma with dentinoid, which is an odontogenic carcinoma that has not yet been fully recognized as a unique entity, has also been shown to harbor pathogenic mutation in *CTNNB1* and *APC* [81]. Although these genes are not part of the MAPK/ERK pathway, they belong to WNT/β-catenin, which is well known to cross-talk with MAPK/ERK. Inactivating *APC* mutation and *CTNNB1* activating mutation leads to strong and aberrant cellular β-catenin accumulation [37, 38, 78, 81], and such phenomena have been observed in some odontogenic epithelial tumors, including ameloblastoma and odontogenic carcinoma [78, 81].

Clear cell odontogenic carcinoma has also been shown to harbor *BRAF* p.V600E [10]. However, since a single sample was screened, future studies including a larger cohort of samples may clarify if such mutations are frequent in this tumor.

### Adenomatoid Odontogenic Tumor

An adenomatoid odontogenic tumor is a benign epithelial odontogenic tumor predominantly affecting the maxilla. It is often encapsulated and has an indolent clinical behavior [1, 82]. Adenomatoid odontogenic tumors most often occur sporadically. However, multiple adenomatoid odontogenic tumors can occur in patients with Schimmelpenning syndrome (OMIM#163200) [83, 84]. This syndrome is caused by postzygotic mutations in *RAS* genes [85]. Based on that knowledge, we screened an adenomatoid odontogenic tumor sample from a Schimmelpenning syndrome patient as well as two sporadic cases for mutations in a NGS panel comprising 50 tumor suppressor genes and oncogenes, including *RAS* family genes, by using Ion AmpliSeq^TM^ Cancer Hotspot Panel v2 (Life Technologies, Carlsbad, USA). The *KRAS* c.35G > T mutation leading to p.G12V was detected in all three samples, and it was further interrogated in additional samples. The mutation was detected in 78% (7/9) of tumors evaluated [11].

Following the aforementioned study, we assessed *KRAS* codon 12 mutations by allele-specific qPCR and screened codons 12, 13, and 61 by Sanger sequencing in a larger cohort of samples. We detected recurrent *KRAS* p.G12V (*n* = 15/38) or p.G12R mutations (*n* = 12/38) in 27 out of 38 adenomatoid odontogenic tumors (71%) regardless of the age of the patient, tumor size, tumor location, follicular or extrafollicular variants, and fibrous capsule thickness [12]. Confirming our findings, the study of Bologna-Molina et al. also reported *KRAS* codon 12 mutations in 78% (7/9) of cases, with either p.G12V (*n* = 2/9) or p.G12R (*n* = 4/9) mutations reported in 6/9 samples and p.G12D in a single case (1/9) [74].

Further than investigating point mutations, we have also investigated the copy-neutral loss of heterozygosity (cnLOH), as well as copy number alterations (CNAs) in a sporadic adenomatoid odontogenic tumor and a sample from a Schimmelpenning syndrome patient [11]. The sporadic tumor showed two rare CNAs, being one at 6p15 and the other at 7p15.3, covering the *IGF2BP3* gene, while the tumor from the syndromic patient only harbored common gains and losses [11]. The deletion only encompasses an intronic portion of the gene, and the significance of these findings in the context of tumorigenesis is unclear.

While the *BRAF* p.V600E mutation emerged as a molecular signature for ameloblastomas and has also been shown to be frequent in other tumors with ameloblastic differentiation, *KRAS* codon 12 mutations seem to be a marker of adenomatoid odontogenic tumors [11, 12, 74]. Even tumors wild-type for *KRAS* showed MAPK/ERK pathway activation, demonstrated by the immunoexpression of the surrogate marker ERK1/2 phosphorylated form [12]. Therefore, whole-exome sequencing of the *KRAS* wild-type cases may provide information on the genetic signatures of the remaining 30% of cases for which *KRAS* mutations have not been detected.

## Molecular Pathology and Targeted Therapy

Although the molecular pathogenesis of ameloblastomas has not been entirely elucidated, the high frequency of *BRAF* p.V600E mutation strongly supports such mutation as a therapeutic target in a large proportion of these tumors. The *BRAF*-mutant human cancers, e.g., melanomas, show a good response to BRAF-targeted inhibition [86]. In line with that, *in vitro* studies and case reports have focused on BRAF-targeted therapy in ameloblastomas [51, 52, 87–92]. Notably, ameloblastoma cell lines harboring *BRAF* p.V600E mutation seem to be sensitive to vemurafenib, i.e., a BRAF small molecule inhibitor, which inhibited cell proliferation and MAPK/ERK activation [51, 52]. Reduction in the tumor mass has also been reported by using dual therapy with BRAF/MEK inhibitors, i.e., dabrafenib and trametinib, in metastatic ameloblastomas [87, 91] and monotherapy, i.e., dabrafenib or vemurafenib, in recurrent [88–90] and metastatic [92] ameloblastomas.

Despite the absence of recurrent MAPK/ERK pathogenic mutations in odontogenic myxomas [93], a pilot study demonstrated positive immunoexpression of ERK1/2 phosphorylated form suggesting MAPK/ERK pathway activation in odontogenic myxoma [94]. Moreover, in this study, MEK was investigated as a possible therapeutic target using the administration of U0126 (a MEK inhibitor), showing promising results both in *in vitro* and *in vivo* tests, which expresses a possible potential use of MEK inhibitors in aggressive cases of odontogenic myxomas [94]. However, considering the limitation of this pilot study, including limited sample numbers, such insights need further confirmation by additional reports.

It is worth noting that the successful results of the above-mentioned studies suggested that molecular targeted therapy is potentially a neoadjuvant treatment and could diminish morbidities related to radical surgery in aggressive and advanced cases of odontogenic tumors carrying MAPK/ERK activating mutations [95]. Nonetheless, the use of MAPK-targeted therapy is still debatable since conservative approaches have demonstrated a high recurrence rate and the use of BRAF/MEK inhibitors might lead to the development of drug resistance and/or serious adverse effects [96, 97]. Further clinical trials addressing the use of BRAF, BRAF/MEK, and MEK inhibitors for odontogenic tumor treatment are necessary to clarify their effectiveness, the advantages and disadvantages, actual efficacy, and best treatment regimen for the patients. Therefore, molecular targeted therapy should not be applied in all MAPK-related odontogenic tumor cases but should be reserved for the most aggressive ones as the first step of treatment to reduce morbidity.

## Concluding Remarks

There is still very limited information on the molecular pathogenesis of odontogenic tumors, with most of the studies focusing either on single genetic mutations or in small gene panels. The advances in molecular techniques for mutation screening have allowed a better understanding of the molecular basis of odontogenic tumors during the last decade. Although their pathogenesis has not been entirely elucidated, the detection of pathogenic mutations in MAPK/ERK pathway genes in some odontogenic tumors strongly suggested that this pathway constitutive activation contributed to tumorigenesis. In a physiological context, the MAPK/ERK signaling pathway regulated important cellular biological functions and plays a role in odontogenesis. Therefore, disturbances in this signaling pathway can lead to odontogenic tumor development.

Among the MAPK/ERK mutations reported in odontogenic tumors, the most prominent is *BRAF* p.V600E. This mutation is present in a high percentage of ameloblastomas, including conventional (64%), unicystic (81%), and peripheral (63%) variants, being considered a molecular signature for this locally aggressive odontogenic tumor. In addition, *BRAF* p.V600E has also been reported in benign mixed odontogenic tumors, namely, ameloblastic fibroma, ameloblastic fibrodentinoma, and ameloblastic fibro-odontoma, and in malignant odontogenic tumors, namely, ameloblastic carcinoma, ameloblastic fibrosarcoma, and clear cell odontogenic carcinoma. Importantly, *KRAS* p.G12V or p.G12R mutations have been reported in ~70% of adenomatoid odontogenic tumors, an encapsulated and indolent epithelial odontogenic tumor. Therefore, the constitutive activation of the same MAPK/ERK pathway in odontogenic tumors with such distinct biological behaviors emphasizes that the functional effects of the pathogenic mutations that disrupt the pathway are context-dependent.

The identification and clarification of the role mutations in MAPK/ERK components play in odontogenic tumors is of core importance for translational application in this heterogeneous and complex group of lesions. Targeted therapy may be a useful tool in the treatment of aggressive and advanced odontogenic tumors. Although targeted therapy has mainly focused on ameloblastomas, further *in vitro* and *in vivo* studies, and clinical trials may help to establish standardized therapeutic regimens both for ameloblastomas and other *BRAF* mutation-positive odontogenic tumors. MAPK/ERK cross talks with other signaling pathways must also be considered in studies focusing on targeted therapy.

Besides being important for therapeutics, the elucidation of *BRAF* and *RAS* mutations in odontogenic tumors might be helpful to diagnosis and classification. For instance, molecular screening can help to solve cases with challenging diagnoses. Moreover, the discovery of *BRAF* p.V600E mutation in ameloblastic fibromas, ameloblastic fibrodentinomas, and ameloblastic fibro-odontomas and the absence of such mutation in odontomas suggested that at least a subset of these tumors should not be classified as the early stages of odontoma.

Currently, these MAPK signaling pathway mutations are known to occur in specific odontogenic tumors, but the question is whether they are important in the initiation of these tumors. Furthermore, whether these mutations impact tumor evolution remains a question. The activation of the MAPK/ERK pathway is linked to cell senescence in other tumorigenic processes. This leads to the question if the same hold true for odontogenic tumors. Moreover, why ameloblastomas show a higher frequency of *BRAF* mutation than their malignant counterpart, ameloblastic carcinomas, remains a question. Although several odontogenic tumors have recently been included in the spectrum of MAPK/ERK-driven tumors, further studies are needed to clarify the role such mutations play in the pathogenesis of odontogenic tumors.

## Author Contributions

CCG provided leadership for the project. LMG, BPC, and CCG performed data/evidence collection and analysis, wrote the initial draft, and generated the text. LPG elaborated the figures. CCG and RSG critically revised the manuscript. All authors contributed to the final manuscript and had final approval of the submitted version.

## Funding

This study received funding from The Coordination for the Improvement of Higher-Level Education Personnel (CAPES)/Brazil [finance code 001], Fundação de Amparo à Pesquisa do Estado de Minas Gerais (FAPEMIG)/Brazil and The National Council of Scientific and Technological Development (CNPq)/Brazil. LMG and BPC receive CAPES and CNPq scholarships, respectively. RSG and CCG are research fellows at CNPq.

## Conflict of Interest

The authors declare that the research was conducted in the absence of any commercial or financial relationships that could be construed as a potential conflict of interest.

## Publisher's Note

All claims expressed in this article are solely those of the authors and do not necessarily represent those of their affiliated organizations, or those of the publisher, the editors and the reviewers. Any product that may be evaluated in this article, or claim that may be made by its manufacturer, is not guaranteed or endorsed by the publisher.
